# The collateral damage of the COVID-19 pandemic on surgical health care in sub-Saharan Africa

**DOI:** 10.7189/jogh.10.020347

**Published:** 2020-12

**Authors:** Kathryn Chu, Ché L Reddy, Emmanuel Makasa, for the AfroSurg Collaborative, Bruce Biccard, Abebe Bekele, Sean Chetty, Edward Clune, Lucia D’Ambrusio, Justine Davies, Rowan Duys, Khumbo Jere, Patrick Kamalo, Susan Levine, Edwin Lugazia, Salome Maswime, Godfrey Muguti, Shingai Nyaguse, Shrikant Peters, John Tarpley, Margaret Tarpley, Anudari Zorigtbataar

**Affiliations:** 1 Centre for Global Surgery, Department of Global Health, Stellenbosch University, Stellenbosch, South Africa; 2 Program in Global Surgery and Social Change, Department of Global Health and Social Medicine, Harvard Medical School, Boston, Massachusetts, USA; 3 Department of Plastic and Oral Surgery, Boston Children’s Hospital, Boston, Massachusetts, USA; 4 Wits Centre of Surgical Care for Primary Health and Sustainable Development, School of Clinical Medicine, University of Witwatersrand, Johannesburg, South Africa

The COVID-19 pandemic has swept across the globe at an unprecedented pace. The first COVID-19 case arrived in Sub-Saharan Africa (SSA) on February 28, 2020, and there are over 600 000 cases spread across the continent [1]. The World Health Organisation has predicted up to a quarter of a billion infections on the continent [2]. In preparation, SSA countries have sharply down-scaled non-COVID-19 health services, including emergency and essential surgical health care (EESC). However, surgical conditions contribute up to a third of the global burden of disease [3]. Surgical health care services are therefore essential to address common conditions that affect mothers, children and adults throughout their lifespan; yet most people in the world (an estimated 5 billion) cannot access such essential care. Scaling down EESC in SSA is likely to have significant and enduring health consequences for the region. Surgery is a vital component of health care services needed to achieve the health priorities in SSA. Several of these priorities are articulated in the Sustainable Development Goals (SDGs) and regional intergovernmental entities [4], and include maternal and child health, injuries and non-communicable diseases. With recent estimates suggesting that postoperative deaths are the third-highest cause of death, globally [5], quality is a significant consideration [6], in addition to expanding access in SSA. However, women are 50 times more likely to die from caesarean sections in SSA compared to their counterparts in high-income countries [7]. Expanding access, in addition to improving the quality of surgical care is, therefore, a requisite for SSA nations to attain health targets in maternal and child health, cancer, injuries and universal health coverage. Before COVID-19, SSA nations were amongst the countries with the most limited access to surgical health care globally [3]; with hindsight, the current pandemic could very well be the “straw that broke the camel’s back”, requiring a much harder restart, more significant investment, time and commitment. Safe, timely, and affordable surgical health care is considered a core element of health service delivery, with significant benefits for broader economic growth and sustainable development in SSA [8]. In this paper, we discuss how health system changes due to COVID-19, in particular the preparedness response, are increasing the barriers to EESC in SSA.

## COVID-19 PANDEMIC IS INCREASING THE BARRIERS TO EESC

The COVID-19 pandemic has disrupted surgical health care delivery in SSA by reducing surgical delivery, interrupting surgical training, and undermining the political and research priority of surgical health care amidst other global health challenges.

Access to EESC is a crucial component of universal health coverage (UHC). A recent modelling study concluded that 866 449 procedures in SSA would be cancelled or postponed during the peak 12 weeks of disruption due to COVID-19, including 737 967 benign, 82 037 cancer and 46 445 obstetric operations [9]. Heightened barriers will result in an increase in avoidable morbidity and mortality due to common EESC, including traumatic injuries, burns, advanced cancer, lower limb ischemia from poorly managed diabetes, and other consequences of untreated conditions in subsequent months.

**Figure 1:**
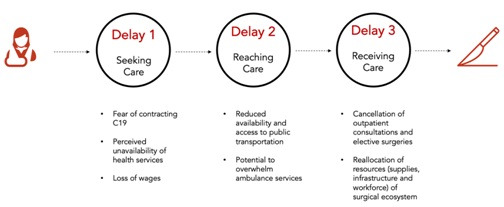
Potential delays in accessing surgical care in Sub-Saharan Africa due to COVID-19.

The barriers to access EESC can be understood through the *Three Delays Framework,* which classifies barriers into seeking, reaching, and receiving care (**Figure 1**) [3]. The fear of contracting COVID-19 from a health facility and the lack of public awareness of available non-COVID-19 health services during lockdown could prevent persons from seeking surgical health care. Many have lost formal and informal employment, and those with surgical health conditions may not have the financial resources to seek or receive care [10]. There are increased barriers to reaching surgical health care due to a lack of public transport services during lockdown periods. Patients depend on public transport to travel to hospitals since pre-hospital emergency medical services, including the availability of ambulances, are severely limited in SSA [11]. Barriers to receiving surgical health care (for instance, life-saving caesarian sections or cancer resections, limb-salvaging management of fractures, or neonates born with congenital anomalies) have increased due to health system changes in service delivery and the allocation of resources. To reduce hospital admissions, facilities have cancelled both outpatient consultations and elective operations. Operating theatres and inpatients wards – indispensable resources of the surgical ecosystem – have proven to be indispensable assets for COVID-19 management, and have been consequently, repurposed [12].

## CHALLENGES TO ENSURE ACCESS TO EESC DURING COVID-19 IN SUB-SAHARAN AFRICA

Timelines to re-escalate surgical health care and address backlogs are unclear, as the COVID-19 surge in SSA is behind that of Asia, North America, and Europe. The American College of Surgeons (ACS) outlined criteria for the re-escalation of surgical operations which include decreasing new COVID-19 cases daily for two weeks, availability of pre- and postoperative testing, and procurement of a surplus of personal protection equipment (PPE) to prepare for a second COVID-19 wave [13]. However, these guidelines may not be appropriate for surgical health care services in SSA. Early data from COVID-19 infected surgical patients have demonstrated higher morbidity and mortality compared to non-COVID-19 patients which makes resuming high-quality surgical health care challenging during the pandemic [14]. COVID-19 testing in SSA remains limited and current capacity would not allow for responsive increases in preoperative and postoperative testing. Results for current COVID-19 tests in some SSA countries take over a week making preoperative testing ineffective for assessing risk [15]. PPE is in meager supply and prioritised for health care workers treating COVID-19 patients.

**Figure:**
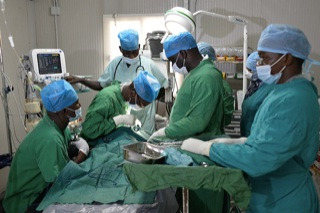
Photo: From Wikimedia (http://bitly.ws/9hrH).

Surgeons, obstetricians, and anaesthesiologists (SOA) are scarce resources in SSA, and their training has been undermined. Due to the shortage of training programs in their home countries, many SSA physicians pursue surgical training in other African countries (South Africa, for instance, is a common training ground [16]), Europe, North America, or Australia. However, training has been adversely affected in terms of operative exposure, teaching, research, examinations, and funding [17] – all of which are essential aspects to their specialisation and which will curtail the quality and duration of their training. With many SOAs redeployed to the frontline and make personal sacrifices, ensuring their safety and well-being is essential to their protection and continued enthusiasm to pursue their demanding surgical training programmes.

Recent political efforts in 16 southern African countries through the Southern African Development Community, an inter-governmental platform and regional economic zone of the African Union, have established the importance of EESC as regional health and economic priorities, and as significant components of attaining UHC and the Sustainable Development Goals (SDGs) [18]. The COVID-19 pandemic threatens to disrupt their commitment due to changes in funding flows and government national health priorities.

Finally, research conducted in SSA is needed to inform and translate political support into evidenced-based surgical health care programs that will strengthen health systems based on local specificities. The COVID-19 pandemic has halted surgical research, routine data collection, and quality assessment and improvement. Academic global surgery, a nascent field in global health, is a crucial component of strengthening surgical health care in SSA. Global surgery partnerships are a gateway to additional funding for academic and policy stakeholder meetings, and research to support national health ministries in their efforts to improve surgical delivery. Much of these collaborative efforts have been curtailed [19].

## MITIGATING THE DAMAGE

The maintenance of surgical health care services for EESC during the COVID-19 pandemic should be prioritised. First, we stand behind the call for economical and accurate rapid COVID-19 tests for SSA in order to conduct pre- and postoperative patient and staff testing as needed. Second, we support strengthening and expansion of anaesthesia and critical care, crucial components of surgical health services, as part of comprehensive COVID-19 emergency preparedness and response for the evolving COVID-19 surge in SSA. This expanded capacity can be used post-pandemic to improve the quality of surgical health care as a component of UHC and for SDG attainment. Third, we call upon the professional associations, together with academic heads of surgical departments to rapidly develop, implement and scale-up measures that both protect surgical trainees and create an enabling environment for their continued learning and retention within the public sector training programs. Finally, we encourage global surgery academic collaborative partnerships to continue through online platforms to share challenges and solutions throughout SSA on how to deliver surgical health care for emergency and essential surgical conditions during the COVID-19 pandemic.

## CONCLUSION

Despite a huge and evolving unmet surgical need in SSA – and the indispensable role of surgery to attain SSA health targets – surgical health care services in SSA are amongst the most inadequate globally in access and quality. The collateral damage of COVID-19 pandemic on surgical health care in SSA will compound an already dire state and are likely to produce enduring consequences for population health, the economy and broader sustainable development; these consequences will be difficult to reverse. The additional barriers to access to care for EESC could result in a tsunami of operative needs in the coming months and increases in avoidable morbidity and mortality. Efforts to increase the surgical workforce have been diminished through limitations in training. Funded research through international collaborative networks to strengthen fragile surgical health care systems and political prioritisation have also been interrupted. We must continue to advocate for equitable access to surgical health care during this unprecedented time, or an excess of lives will be lost.
